# Ethnobotanical knowledge of the lay people of Blouberg area (Pedi tribe), Limpopo Province, South Africa

**DOI:** 10.1186/s13002-018-0245-4

**Published:** 2018-07-13

**Authors:** Nkoana Ishmael Mongalo, Tshepiso Jan Makhafola

**Affiliations:** 1 College of Agriculture and Environmental Science (CAES) Laboratories, University of South Africa, Private BagX06, Johannesburg, 0710 South Africa; 2 Research, Innovation & Engagements Portfolio, Mangosuthu University of Technology, P O Box 12363, Durban, 4026 South Africa

**Keywords:** Blouberg area, Limpopo Province, Ethno-medicine, Ethnobotanical urvey, Medicinal plants, South Africa

## Abstract

**Background:**

Limpopo province, South Africa, has a rich plant diversity and is referred to as one of the hotspots areas within the country. The aim of the current work was to identify and document medicinal plant species used by the indigenous Pedi people of Blouberg area, Limpopo Province, South Africa.

**Methods:**

A total of 40 informants which includes both traditional healers and medicinal plant sellers were randomly selected and asked about the plant species used in treatment of variety of infections using a structured questionnaire. Follow-up visits and various field walks were also used to identify and document various plant species used in Traditional medicine (TM). The interviews were carried out from April 2008 to June 2016 using indigenous language (Sehananwa).

**Results:**

A total of 82 medicinal plants species belonging to 42 families have been collected, identified and documented. About 46.34% of the plant species were herbs, followed by trees (25.61%), shrubs (20.73%) and climbers (7.32%). The most used plant parts are roots and rhizomes (58.58%). *Peltophorum africanum* Sond revealed frequency index of greater than 70 and is used in combination with other plants species to treat various pathogenic infections. Most of the plant species reported are used in the treatment of sexually transmitted infections (24), management of HIV-AIDS (15) and stomach ache (14). Our informants indicated that the use of plant medicines in combinations is also applied to cure pathogenic infections.

**Conclusion:**

The current study demonstrate that the indigenous people of Blouberg area, Limpopo Province harbours an important information about the vegetation around them. The plant species are used in the treatment of various pathogenic infections, offers fruits as additional source of food and form integral part of other medicinal products that may in turn produce income.

## Background

Limpopo Province is mostly dominated by the Pedi (57%), Tsonga (23%) and Venda (12%), ethnic groups while English and Afrikaans speaker only constitutes less than 4% combined [1]. However, there are other unofficial languages which includes Khelobedu, Setlokwa and Sehananwa falling under the Sotho or Pedi speaking people. Blouberg area, dominated by Pedi tribe, comprise of only two main health care facilities (Blouberg and Helena-Franz Hospital), a small remote town known as Senwabarwana and a few game reserves (Blouberg and Maleboho nature reserves). The population in this area, like in other rural African communities is reliant on traditional medicine (TM) as their basic source of health care [2, 3]. The other possible challenges in health care facilities within the study site may include long distances travelled to hospitals, long waiting on the queues, drug shortages, lack of proper laboratories with state of the art scientific equipment and attitudes of the health workers [4]. The area is one of the medicinal plants hotspots with only little plant species documented in the few surveys taken recently within the Province [5–9], but not strictly focussing on Blouberg area.

Several ethnobotanical studies have been taken world-wide, documenting different plant species and preserving the indigenous knowledge of various communities [10–15]. Most of these surveys may well serve as possible leads for the discovery of potent new drugs that may be used to combat most harmful infections that pose a serious threat to human and animal health. Traditional people believe in using TM or herbal therapy in treating various infections, mostly because plant species are abundant in nature in their surrounding environment, less priced and are believed to pose less or no side effects. Moreover, it is believed that herbal therapy is holistic, integrating the emotional, spiritual and mental well-being of the patients [16]. Furthermore, TM is culturally acceptable and there is a belief that it purges out any infection after treatment from hospitals [17]. Besides being the main source of drugs in the current threatened health care system with emerging multiple resistant organisms, the traditional medicine still receives little attention world-wide [18].

The enormous rise in HIV-AIDS infections in Africa pose a further threat to human life, resulting in variety of opportunistic infections which may include various skin infections inflammatory disorders, various forms of candidiasis, reactivation of the TB germ and other possible pulmonary infections, multiple forms of lymphoma and various Herpes infections [19–21]. The aim of the current work is to identify and document various plant species used by the lay people of Blouberg (Hananwa).

## Methods

### Study area

**Fig. 1:**
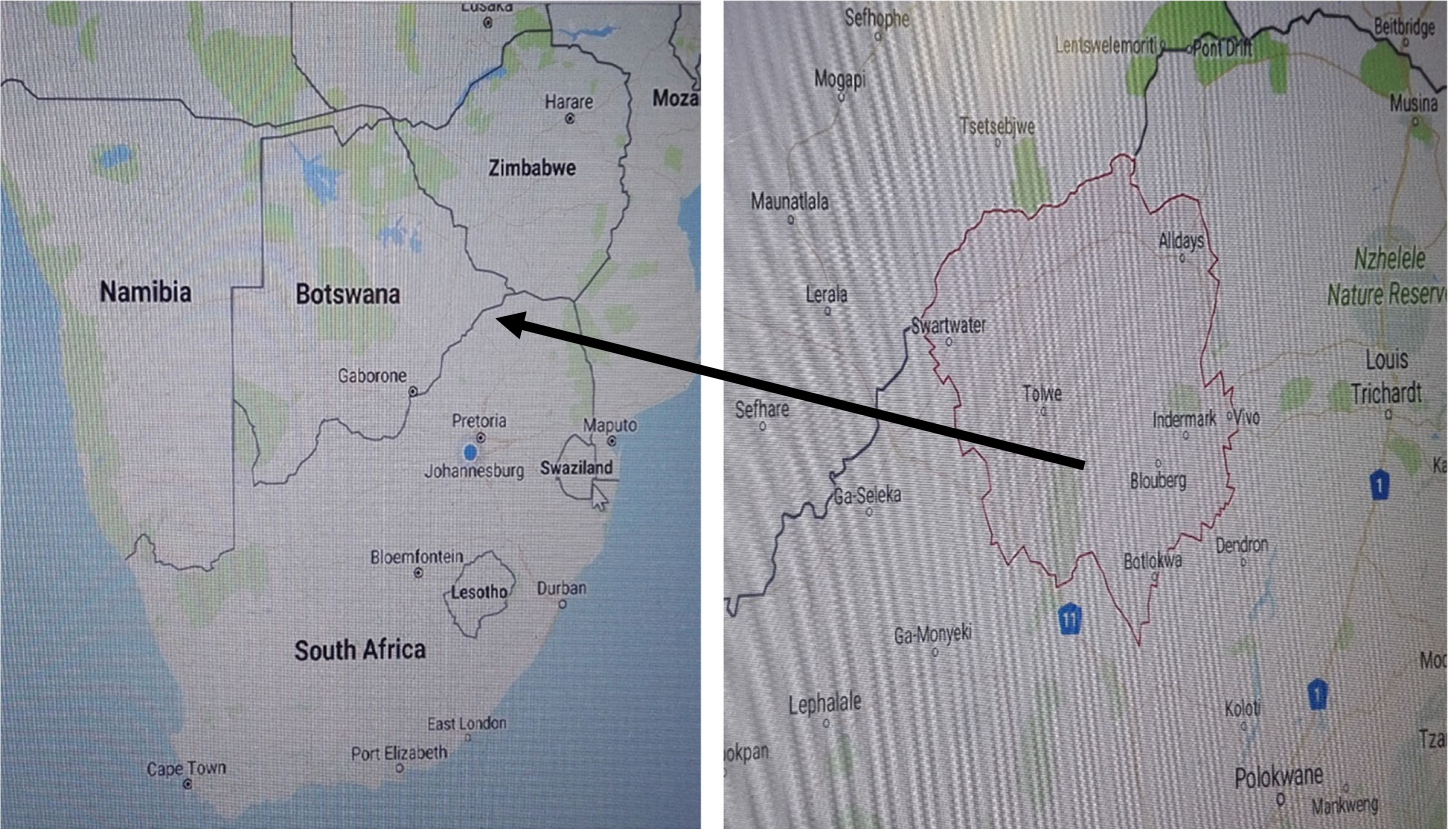
Map of South Africa, locating Blouberg area (red coloured area)

South Africa (Fig. 1) is divided into nine Provinces. Blouberg area, indigenously known as Hananwa, is situated in the Limpopo Province, 30 km north of Dendron and 95 km from Polokwane, and connects South Africa to both Botswana and Zimbabwe. Geographically, it is a deep rural area, mountainous and located between the Waterberg Wetlands and the Dongola Trans-frontier and extends right up to the Botswana border [22].

The area is under kingship of the Leboho family and occupies an area of approximately 5054 km^2^ and a total population of about 166,243 people [23]. Big rivers such as Bohlokwe, Tswatšane, Mmatšope and other small rivers provide water to various livestock in the area. Mogalakwena River, with its rich biota which includes crocodiles, also cuts into the area. Various sites on the mountain Blouberg- a green mountain throughout the year, serves different purposes. Ploughing fields, burial site for the kings of the past and hunting grounds are amongst the most important sites in the mountain.

### Selection of informants and interviews

A total of 40 informants which includes 20 traditional healers and 20 medicinal plants sellers from Blouberg area have been randomly sampled from a pool of informants attending meeting relating to African Dingaka Association of South Africa at Blouberg area from April 2008 to June 2016 using structured questionnaires, follow-up visits and field trips. Various visits were made to establish a rapport and more often assisting in plant collection for use in the African surgeries (consulting rooms). Informants were mostly from villages such as Lethaleng, Ga-Mochemi, Ditatšu, Ga-Rammutla A, Pickum B, Ga-Mashalane, Ga-Mmamolele, Ga-Broekmane, Dilaeneng, Ga-Machaba, Ga-Kibi, Ga-Mmaleboho, Ga-Radimang, Ga-Manaka, Sewale, Bosehla, Mohlabeng-wa-Malokela, Gwarung, Ga-Kobe, Sebotlane, Ga-Kibi, Devrede, Makgabeng, Marobjane, Inveraan and Bull-bull. The informants agreed to furnish information regarding the indigenous medicinal plant species used in the treatment of various infections treated by both healers and plant sellers using questionnaires, while noting the plant species named using Sehananwa as a local language. At a later date, informants were requested to identify the plant species at various collection sites.

Follow-up visits were later conducted with the intention of verifying the information given by informants, particularly the correspondence of indigenous names, and then later request further information on whether the plant species mentioned are used as a single plant material or in combination with other plant species. Only plant species mentioned by the informants at least three times were eligible for documentation [24]. Data on combinations of plants in the treatment of infections was documented, including mode of preparation and route of administration.

### Collection and identification of medicinal plants

The plant species were collected, pressed and identified by botanists in the Department of Life and Consumer Sciences, Horticulture and College of Agriculture and Environmental Science (CAES) laboratories. The unknown plant materials to the authors and staff in the College of Agriculture and Environmental Sciences were send to National Botanical Institute (NBI) in Pretoria for identification (The personnel who assisted in identification includes Klopper, R.R., Mothogoane, M.S., Makgakga, M.C., Makwarela, L.E., Archer, R.H., Nkonki, T., Ready, J.A., Bester, S.P., Meyer, J.J., Ruiters, A.K. and Welamn, N). The voucher specimen of all the collected plant species were then lodged at University of South Africa herbarium in Florida. Other plant materials were also deposited into the NBI herbarium. These includes *Cissus cornifolia*, *Neorautanenia mitis*, *Pollichia campestris*, *Ipomoea albivenia*, *Pterodiscus kellerianus*, *Ehretia rigida* and *Aptosimum lineare*.

### Data analysis

The analysis of data was carried out using both descriptive and inferential statistics using percentages and frequencies. The frequency index (FI), informant consensus factor (F_ic_) and Fidelity levels (FL) were calculated and compared. FI of the documented plant species were calculated using the formula:

$$ \mathrm{FI}=\mathrm{FC}/N\kern0.5em \times 100, $$

Where FI is the frequency of citation for one plant species by informants, FC is the number of informants who cited the use of the plant species and *N* is the total number of informants [25, 26]. F_ic_ was calculated to determine the homogeneity of the information provided by the informants using the formula:

$$ \mathrm{FIC}={\mathrm{N}}_{\mathrm{ur}}\hbox{-} {\mathrm{N}}_{\mathrm{taxa}}/{\mathrm{N}}_{\mathrm{ur}}\hbox{-} 1 $$

Where N_ur_ is the number of use reports, N_taxa_ is the number of species in each use category [27], while FL was calculated using the formula:

$$ \mathrm{FL}={\mathrm{I}}_{\mathrm{p}}/{\mathrm{I}}_{\mathrm{u}}\mathrm{X}\ 100 $$

Where I_p_ is the number of informants who suggested the use of the species for the same major ailment and I_u_ represents the total number of informants who mentioned the species for any use [28].

## Results

### Socio-demographic information and diversity of plants species

The communities around Blouberg area use diverse flora in treatment of various ailments and local people possess a rich traditional knowledge on the use of medicinal plants as medicine. The age of our informants ranged from 30 to 88 years (Table 1). About 64% of our informants are aged between 40 and 65 years of age while 10% of our informants are below the age of 40 years. About 40% of our informants have never been to school and only one of the 40 participants possess a diploma in Education and is also a well-known traditional healer. A total of 82 plant species belonging to 42 different families were recorded in the current study (Table 2). Families such as Fabaceae (14.63%), Malvaceae (8.54%), Apocynaceae (7.32%), Solanaceae (6.10%), Convolvulaceae (4.88%), Euphorbiaceae (3.66%) and Vitaceae (3.66%) were well represented (Table 3) and are dominant, while families such as Rubiaceae, Olacaceae, Loganiaceae, Ebenaceae, Celastraceae, Asphodelaceae and Anacardiaceae reported 2.44% each. The other families recorded one plant species each.

**Table 1:** Demographic of informants

Informants category	Males	Females	Age groups				Level of Education				
			30–40	41–50	51–65	Above 65	Never been to school	ABET Education	Primary Education	Secondary Education	Tertiary Education
Traditional healers	8	12	–	1	9	10	6	4	3	6	1
Plant sellers	14	6	4	12	4	–	10	0	2	8	0
Percentage	55	45	10	32.5	32.5	25	40	10	12.5	35	2.5

**Table 2:** Ethnobotany of the Pedi tribe of Blouberg area, Limpopo Province, South Africa

Family/Voucher number	Plant species	Growth form	Plant part used	Indigenous name(s)	Ethno-medicinal uses	Frequency Index	Mode of administration
Acanthaceae							
MNI-18	*Blepharis diversispina* (Nees) C.B.Clarke.	Herb	Roots	Mookapitsi	Roots are used to treat the deceased’s wife and sexually transmitted infections.	73	Nasal
Amaryllidaceae							
MNI-81	*Ammocharis coranica* (Ker Gawl.) Herb.	Herb	Bulb	Mmotu wa fase	Bulb is used in the treatment of foot ache.	23	Fresh pieces of bulb is cooked and resulting solution is applied directly to affected area.
Anacardiaceae							
MNI-82	*Mangifera indica* L.	Tree	Stem bark	Mo-Mango	Stem bark is used to treat heart infections and diarrhoea	33	Oral
			Fruits		The fruits are edible		
MNI-17	*Sclerocarya birrea* (A.Rich.) Hochst.	Tree	Stem bark	Morula	Stem bark is used to treat sexually transmitted infections, a general immune booster for HIV-AIDS patients and as blood purifier. Stem bark is also used to treat ethno-veterinary infections in cattle.	75	Oral
			Fruits		Fruits are edible and may be used to prepare home-made beer.		
Apiaceae							
MNI-20	*Peucedanum sulcatum* Sond.	Herb	Roots	Mongamo	Roots are used as general medicine	28	Oral.
Apocynaceae							
MNI-30	*Carissa edulis* (Forssk.) Vahl.	Shrub	Roots	Mothokolo	Roots are used to treat sexually transmitted infections	33	Oral
			Leaves		Sap from the leaves is used to treat sores and wounds from the body.		Sap and chopped fresh leaves are immersed in hot water overnight and then used to wash wounds
			Fruits		Fruit are edible		
MNI-41	*Catharanthus roseus* (L.) G.Don	Herb	Roots	Lepolomo	Roots are used to treat skin related infections and “dropsy” a sexually transmissible disease.	30	Oral and topically applied to affected area.
MNI-39	*Nerium oleander* L.	Shrub	Leaves	Five-roses	Leaves are used to treat tooth ache.	10	Fresh leaves are chopped, immersed in water overnight and used to rinse the mouth.
			Roots		Roots are used to treat diarrhoea.		Oral
MNI-49	*Sarcostemma acidum* (Roxb.) Voigt	Climber	Whole plant	Moraro	Whole plant used for magical purposes.	58	Blown
MNI-50	*Sarcostemma torreyi* (A. Grey) Woodson	Climber	Whole plant	Moraroana	Whole plant used for magical purposes.	30	Blown
MNI-33	*Raphionacme hirsuta* (E.Mey.) R.A.Dyer	Herb	Bulb	Tshengwa	Bulb used to treat sexually transmitted infections and may be carved into a wheel that can be used by boys when playing.	53	Oral
Asparagaceae							
MNI-48	*Asparagus racemosus* Willd.	Herb	Roots	Mophatlalatamaru	Roots are used as food for new-borns	8	Oral, mostly using a bottle for milk.
			Whole plant		Whole plant is used for magical purposes		Burned
Asphodelaceae							
MNI-54	*Aloe zebrina* Baker	Herb	Roots	Tsikele	Roots are used to treat sexually transmitted infections	35	Oral
			Whole plant		Whole plant is used for magical purposes. It is believed to dispel witches when grown in a home, both sides of the gate.		–
MNI-79	*Aloe marlothii* A.Berger.	Shrub	Leaves	Seema ka Maoto	Liquid strained from the leaves is used to treat skin infections including sores and wounds. Leaves are also used to treat ethnoveterinary infections.	15	Topically applied to affected areas.
Asteraceae							
MNI-52	*Geigeria aspera* Harv.	Herb	Whole plant	Makgonatsohle	Whole plant is used to cure various stomach related illnesses.	45	Oral
Boraginaceae							
MNI-44	*Ehretia rigida* (Thumb) Druce *subs. Nervifolia* Retief & A.E. Van Wyk	Shrub	Roots	Mothobethobe	Roots are used to treat new born infections.	18	Oral using a bottle for milk.
			Fruits		Fruits are edible.		
Cactaceae							
MNI-51	*Opuntia ficus-indica* (L.) Mill.	Shrub	Roots	Motloro	Roots are used to treat shingles arising from HIV-AIDS	23	Roots are cooked and resulting liquid is used to wash the sores
			Fruits		The fruits are edible		
Cannabaceae							
MNI-78	*Cannabis sativa* L.	Herb	Whole plant	Motsokomogolo (Patše)	whole plant is used to treat “Vaal sick” and excessive headache.	28	Inhalation
Caricaceae							
MNI-83	*Carica papaya* L.	Tree	Roots	Mophoophoo	The roots are used to treat sexually transmitted infections	25	Oral
			Fruits		The fruits are edible		
Caryophyllaceae							
MNI-40	*Pollichia campestris* Aiton	Herb	Roots	Tshimanenyana	Roots are used to treat HIV/AIDS related infections.	15	Oral
Celastraceae							
MNI-58	*Elaeodendron tranvaalense* (Burtt Davy) R.H.Archer	Tree	Stem bark	Monamane	Stem bark is used to treat sexually transmitted infections.	15	Oral
MNI-85	*Gymnosporia senegalensis* (Lam.) Loes.	Herb	Leaves	Mphato	Leaves are used to treat stomach aches and vomiting.	38	Oral
			Root bark		Root bark is used in the management of HIV-AIDS.		Oral
Combretaceae							
MNI-77	*Terminalia sericea* Burch. ex DC.	Tree	Roots	Monakanakane	Roots are used to strengthen the fontanelle and general immunity of the new born babies.	58	Oral, mostly using a bottle for milk.
			Stem bark		Stem bark is used to treat skin related infections, sexually transmitted infections and opportunistic infections associated with HIV-AIDS.		Oral
Convolvulaceae							
MNI-57	*Ipomoea alba* L.	Climber	Stem bark	Mmolobolo	General medicine	28	Oral
MNI-27	*Ipomoea bolusiana* Schinz	Herb	Bulb	Mokutu	Bulb is used to treat foot ache and sexually transmitted infections	30	Oral, Boiled in water and then applied with a soft cloth to affected leg without wounds.
MNI-84	*Ipomoea spp*	Herb	Bulb	Tlola	General medicine, eaten by boys while shepherding the cows, food for rabbits and medicine for wild animals.	10	Oral
MNI-34	*Ipomoea albivenia* Sweet	Climber	Bulb	Leshilahlole	Bulb is used to treat infertility in women.	15	Oral
Cucurbitaceae							
MNI-36	*Cucumis hirsutus* Sond.	Herb	Roots	Mokapane	Roots are used to treat deceased’s wife.	65	Nasal
			Leaves		Leaves are used to enhance fertility in women.		Oral
Ebenaceae							
MNI-99	*Euclea natalensis* A.DC.	Shrub	Roots	Mokgokgono	Roots are used for magical purposes.	10	Burned
			Fruits		Fruits are edible		
MNI-76	*Euclea undulata* Thunb.	Tree	Stem bark	Mokwerekwere	Stem bark is used is used to treat diarrhoea	50	Oral
			Fruits		Fruits are edible		
Euphorbiaceae							
MNI-59	*Jatropha erythropoda* Pax & K.Hoffm.	Herb	bulb	Thotamadi	Bulb used as blood purifier	25	Oral
MNI-29	*Jatropha zeyheri* Sond.	Herb	Roots	Sefapabadia	Root is used in the treatment of eye infections, gynaecological complaints and sexually transmitted infections. Roots are also used to treat ethno-veterinary infections in cattle.	65	Oral, Roots are immersed in water and used to wash infected eyes daily
MNI-45	*Tragia dioica* Sond.	Herb	Whole plant	Mmabetjane	Whole plant is used to cure sores in the stomach.	20	Oral
Fabaceae							
MNI-60	*Acacia karroo* Hayne	Tree	Roots	Mooka	Roots are used to treat diarrhoea.	15	Oral.
MNI-94	*Bauhinia galpinii* N.E.Br.	Shrub	Roots	Mohohoma	Roots are used to treat sexually transmitted infections	10	Oral
MNI-26	*Cassia abbreviata* Oliv.	Shrub	Roots	Monepenepe	Roots and stem bark are used in the treatment of sexually transmitted infections. Roots are also used to treat mellitus diabetes.	45	Oral
			Stem bark		Stem bark may be used as an aphrodisiac for men, anti-poison and used as a general immune booster for HIV-AIDS patients. Stem barks are used in doctoring of homesteads before the rainy season, preventing the homesteads from lightning.		Oral
			Leaves		Leaves are also used to treat ethno-veterinary infections in cattle.		Oral
MNI-75	*Dichrostachys cinerea* (L.) Wight & Arn.	Tree	Leaves	Moretshe	Leaves are used to treat vomiting, while thorns are used for magical purposes.	18	Oral
MNI-18	*Elephantorrhiza elephantina* (Burch.) Skeels	Herb	Roots	Mohauwane	Roots are used to treat sexually transmitted infections, blood purifier, eye infections and as a general medicine. Roots are also used to treat ethno-veterinary infections in cattle.	85	Oral, Rinsing is applied to eyes after being infused in water overnight.
MNI-21	*Elephantorrhiza burkei* Benth.	Herb	Roots	Mohauwane	Roots are used to treat sexually transmitted infections, blood purifier, eye infections and as a general medicine. Roots are also used to treat ethno-veterinary infections in cattle.	90	Oral
MNI-74	*Erythrina lysistemon* Hutch.	Tree	seeds	Mo-Khupe	Magical purposes.	20	–
MNI-85	*Kirkia acuminata* Oliv.	Tree	Sap from stem bark	Modumela	Sap is used to treat a fractured bone and is believed to accelerate healing. Sap also used for general well-being.	10	Stem is cut and resulting protruding sap is collected dried, ground and applied to fractured bone.
MNI-10	*Peltophorum africanum* Sond.	Tree	Leaves	Mosehla	Leaves are used to treat ethno-veterinary infections in cattle.	78	Oral
			Roots/ stem bark		Roots and stem bark are used to treat sexually transmitted infections, stomach and skin related infections		Oral
MNI-80	*Schotia brachypetala* Sond.	Tree	Whole plant	Molope	Whole plant are used to treat diarrhoea	15	Oral
MNI-42	*Neorautanenia mitis* (A. Rich) Verdc	Herb	Bulb	Letlopya	Bulb is used to treat foot ache	30	Boiled plant material is topically applied to legs
MNI-17	*Urginea sanguinea* Schinz	Herb	Bulb	Sekanama	Bulbs are used to treat sexually transmitted infections and as a blood purifier. Bulbs are also used to treat ethno-veterinary infections.	48	Oral
Hypoxidaceae							
MNI-61	*Hypoxis haemerocallidea* Fisch., C.A.Mey. & Avé-Lall.	Herb	Bulb	Monna wa maledu	Bulb is used as an aphrodisiac for men and used as a general immune booster for HIV-AIDS patients	50	Oral
MNI-42	*Neorautanenia mitis* (A. Rich) Verdc	Herb	Bulb	Letlopya	Bulb is used to treat foot ache	30	Fresh pieces of bulb is cooked and resulting solution is applied directly to affected area topically.
Loganiaceae							
MNI-67	*Strychnos spinosa* Lam.	Tree	Stem bark	Mokwakwa	Stem bark is used to treat diarrhoea and other related infections	20	Oral
			Fruits		Fruits are edible.		
MNI-66	*Strychnos madagascariensis* Poir.	Tree	Roots	Morutla	Roots are used to treat foot ache and mouth ulcers associated with HIV-AIDS.	58	Ground roots are powdered and applied directly on infected area
Malvaceae							
MNI-73	*Adansonia digitata* L.	Tree	Stem bark	Motsoo	Stem bark is used to treat opportunistic fungal infections, mostly associated with HIV-AIDS.	35	Oral
				Fruits	Fruit are edible		
MNI-16	*Azanza garckeana* (F.Hoffm.) Exell & Hillc.	Tree	Stem bark	Motlobya	Stem bark is used to treat painful joints in aged individuals	5	Oral
				Fruits	Fruit are edible		
			Roots		Roots are used to treat heart related and high blood pressure in adults.		Oral
			Fruits		Fruits are edible.		
MNI-24	*Grewia flava* DC.	Herb	Roots	Mothetlwa	Roots are used to cure sexually transmitted infections and excessive diarrhoea.	53	Oral
			Fruits		Fruits are edible and may be collected dried and then mixed with a little mealie meal, cooked into porridge, which may be eaten alone during drought years.		
MNI-62	*Grewia flavescens* Juss.	Herb	Roots	Mopharatshwene	Roots are used as “disha” for the new born.	20	Oral, mostly using a bottle for milk.
			Fruits		Fruits are edible		
MNI-95	*Grewia spp*	Shrub	Roots	Mowana	Roots are used as “disha” for the new born.	23	Oral, mostly using a bottle for milk.
			Fruits		Fruits are edible		
MNI-25	*Waltheria indica* L.	Herb	Roots	Mokhutesela	Roots are used to treat sexually transmitted infections and stomach problems. Also used as food and stomach coolant for new born babies.	70	Oral
MNI-32	*Sida cordifolia* L.	Herb	Whole plant	Mokadi	Whole plant us used to treat high blood pressure	15	Oral
Meliaceae							
MNI-71	*Melia azeadarach* L.	Tree	Leaves	Mosara	Leaves are used to treat infections associated with HIV-AIDS including shingles	30	Chopped fresh leaves are boiled and then liquid used to wash the affected area
Mesembryanthemaceae							
MNI-86	*Carbobrotus edulis* (L.) N.E.Br.	Herb	Leaves	Tima	Leaves are used to treat an STI known as “Tshofela” and may also be used to treat shingles associated with HIV-AIDS.	43	Topically applied to affected area.
Myrtaceae							
MNI-72	*Psidium guajava* L.	Shrub	Roots	Mo-*Guava*	Stomach ache and diarrhoea in adults.	58	Oral
			Fruits		Fruits are edible		
Olacaceae							
MNI-87	*Ximenia caffra* Sond.	Tree	Roots	Motshidikgomo	Roots are used to treat sexually transmitted infections.	30	Oral
			Fruits		Fruits are edible		
MNI-70	*Ximenia americana* L.	Shrub	Roots	Motshidimphiswane	Roots are used in the treatment of asthma, stomach ache and various mouth ulcers associated with HIV-AIDS.	26	Oral, ground fruit is used to wash the ulcers.
			Fruits		Fruits are edible		
Pedaliaceae							
MNI-46	*Pterodiscus kellerianus* Schinz.	Herbs	Roots	Moyane	Fleshy roots are used to treat stomach aches in new-born babies	45	Oral, mostly using a bottle for milk.
Phyllanthaceae							
MNI-56	*Flueggea virosa* (Roxb. ex Willd.) Royle	Shrub	Branches	Mohlakaume	Branches are used for magical purposes.	10	Blown
			Fruits		Fruit are edible		
Poaceae							
MNI-63	*Cynodon dactylon* (L.) Pers.	herb	Whole plant	Mothlakatlhaka	Whole plant may be used to cure tonsils.	8	Grass is boiled in a tin with about 500 ml water and then applied to affected areas.
Polygalaceae							
MNI-69	*Securidaca longipedunculata* Fresen.	Shrub	Root bark	Mphesu	Root bark is used as an aphrodisiac for men	73	Root barks are ground into powder which is taken orally with mageu.
			Root kernel		Root kernel is used to treat Headache		Dried kernels are burned and then inhaled.
Punicaceae							
MNI-88	*Punica granatum* L.	Shrub	Roots	Mokgarenate	Root are used to cure diarrhoea, mostly in HIV-positive patients and other related infections	8	The roots are dried and ground into powder which must be licked by mouth.
				Fruits	Fruits are edible		
Rhamnaceae							
MNI-91	*Ziziphus mucronata* Willd.	Tree	Roots	Mokgalo	Roots are used to treat stomach infections. Roots may also be used to manage HIV and HIDS.	10	Oral
			Leaves		Leaves are used to treat burns and tonsils		Leaves are removed and then chewed by mouth, applied surrounding the affected area
			Fruits		Fruit are edible		
Rubiaceae							
MNI-89	*Gardenia volkensii* K.Schum*.*	Shrub	Branches	Morala	Stem bark is used to treat chest complaints and tuberculosis related infections.	10	Oral
			Stem bark		The branches are cut into pieces which will be mixed with other medicines to doctor homesteads (Magical).		Burned
MNI-64	*Vangueria infausta* Burch.	Tree	Branches	Mmilo	Branches used in doctoring of homesteads	23	Blown
			Fruits		Fruit are edible		
Salantaceae							
MNI-96	*Osyris lanceolata* Hochst. & Steud.	Shrub	Roots	Mphere	Roots are used for magical purposes.	35	Burned
Sapotaceae							
MNI-68	*Mimusops zeyheri* Sond.	Tree	Roots	Monupudu	Roots are used to treat syphilis (sexually transmissible disease), stomach ache and gynaecological infections.	10	Oral
			Fruit		Fruit is edible		
Scrophulariaceae							
MNI-47	*Aptosimum lineare* Marloth & Engl.	Herb	Whole plant	Popeloana	Whole plant is used to treat gynaecological complaints	17	Oral
Solanaceae							
MNI-90	*Solanum aculeastrum* Dunal	Herb	Roots	Morola	Roots are used to treat stomach aches.	30	Oral
MNI-95	*Solanum mauritianum* Scop.	Shrub	Roots	Mothollo	Roots are used to treat stomach aches.	53	Oral
MNI-100	*Solanum panduriforme* E.Mey.	Herb	Roots	Morolana	Roots are used to treat stomach aches.	30	Oral
MNI-93	*Solanum supinum* Dunal	Herb	Roots	Morola	Roots are used to treat stomach aches.	15	Oral
MNI-92	*Withania somnifera* (L.) Dunal	Herb	Roots	Mosalamaropeng	Roots are used to treat infertility and other gynaecological related infections.	35	Oral
Talinaceae							
MNI-35	*Talinum caffrum* (Thumb.) Eckl. & Zeyhr.	Herb	Roots	Peloana	Fleshy harvested roots are used to treat heart related infections.	15	Oral
Vitaceae							
MNI-22	*Cissus quadrangularis* L.	Climber	Whole plant	Mohlabadipoo	Whole plant is used to treat sexually transmitted infections and skin related infections. Stems are also used to treat ethno-veterinary infections in cattle.	73	Both oral and Topically applied to affected area.
MNI-65	*Vitis vinifera* L.	Climber	Roots	Moterebe	Roots are used to treat high blood pressure in adults	12	oral
			Fruits		Fruits are edible.		
MNI-31	*Cissus cornifolia* (Baker) Planch.	Herb	Bulb	Mokgoo	Bulb is used as a general medicine	33	Oral
			Fruits		Fruit are edible		
Xanthorhoeaceae							
MNI-43	*Bulbine angustifolia* Poelln.	Herb	Roots	Marumo a ngata	Roots are used as an aphrodisiac and for general well-being of men	30	Oral

**Table 3:** Plant families with the largest (At least 3 species reported) number of species

Family name	Number of species	Percentage
Fabaceae	12	14.63
Malvaceae	7	8.54
Apocynaceae	6	7.32
Solanaceae	5	6.10
Convolvulaceae	4	4.88
Euphorbiaceae	3	3.66
Vitaceae	3	3.66
Rubiaceae	2	2.44
Olacaceae	2	2.44
Loganiaceae	2	2.44
Ebenaceae	2	2.44
Celastraceae	2	2.44
Asphodelaceae	2	2.44
Anacardiaceae	2	2.44

### Growth forms, plant parts used and mode of administration of plant species

**Fig. 2:**
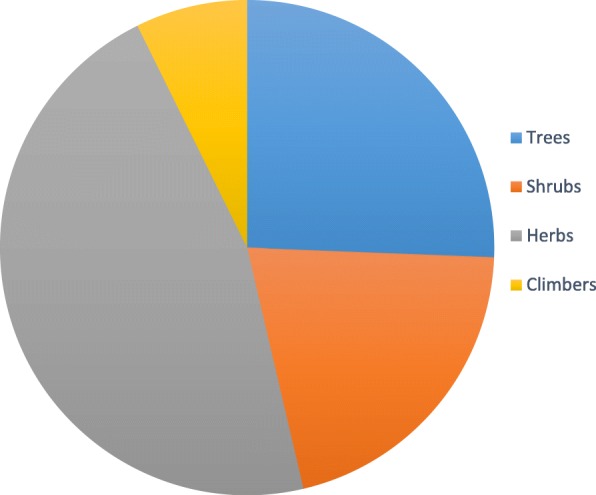
Growth forms of the reported plant species

**Fig. 3:**
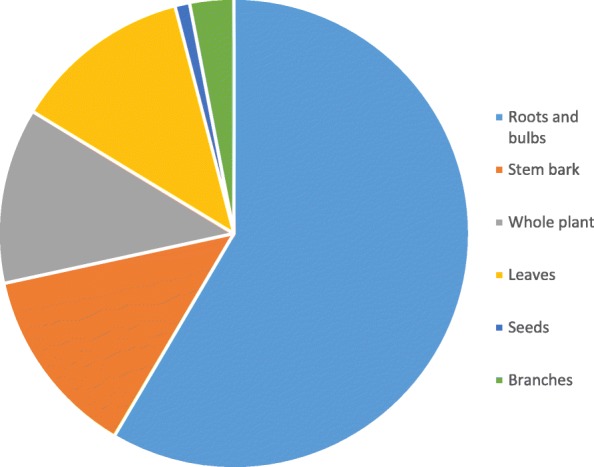
Plant parts used in the study

The reported plant species were dominated by herbs (46.34%), followed by trees (25.61%), shrubs (20.73) and climbers (7.32%) (Fig. 2). Out of the reported plant species, roots and bulbs (underground plant material) were the most used (58.6%), followed by stem bark (13.1%), whole plant (12.1%) and leaves (11.1%) (Fig. 3).

**Fig. 4:**
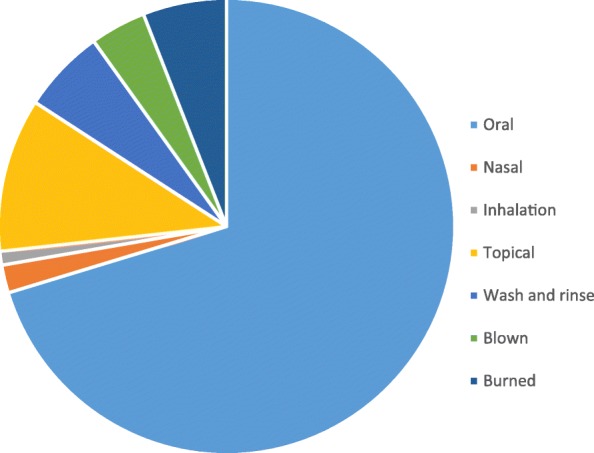
Mode of administration of reported medicinal plants

Most of the plants materials are boiled and taken orally (73%) when treating various types of infections (Fig. 4). The other plant species may be topically applied (10.89%) to the skin, while the others may be burned (5.94%) or used to wash and rinse (5.94%) the infected body part. The inhalation, nasal administration, and plant materials which may be blown reported less than 5% each.

### Ailments treated and consensus agreement

The most reported plant species are used in the treatment of sexually transmitted infections (24) followed by those used in the management of HIV-AIDS related infections (15), stomach ache (14) and plant species used in the treatment of ethno-veterinary infections (9) while the informant consensus factors (F_ic_) of the mentioned ailment categories ranged from 0.78 to 1 as shown in Table 4. About 25 species revealed FL value of 100% against variety of diseases (Table 5).

**Table 4:** Consensus agreement about uses of medicinal plants for important ailment categories

Ailment category	N_taxa_	N_ur_	F_ic_
High blood pressure	3	10	0.78
Joints	1	3	1
Fractured bones	1	4	1
Anti-poison	1	3	1
Aphrodisiac	4	37	0.92
Diabetes	1	3	1
Eye infections	3	30	0.93
Asthma	1	4	1
Tonsillitis	2	6	1
Chest complaints	1	3	1
Gynaecological complaints	6	37	0.86
vomiting	2	8	0.86
Headache	2	13	0.92
Vaal sick	1	7	1
Stomach ache	14	114	0.88
New born infections	7	47	0.88
Diarrhoea	7	43	0.86
Tooth ache	1	2	1
Skin infections	5	28	0.85
Sores and wounds	3	7	0.67
General medicine	6	46	0.89
Ethno-veterinary infections	9	44	0.81
Blood purifier	5	35	0.88
Management of HIV-AIDS	15	110	0.80
Heart infections	3	16	0.87
Foot ache	4	39	0.92
Deceased’s wife	2	49	0.98
Sexually transmitted infections	24	209	0.89
Some taxa falls in more than one ailment categories

**Table 5:** Fidelity levels (FL) of plant species used for various uses by key informants

Medicinal Plant species	Therapeutic uses	I_p_	I_u_	FL %
*Bauhinia galpinii*	Sexually transmitted infections	4	4	100
*Mimusops zeyheri*	Sexually transmitted infections	4	4	100
*Raphionacme hirsuta*	Sexually transmitted infections	16	21	72
*Pollichia campestris*	Management of HIV-AIDS	6	6	100
*Melia azeadarach*	Management of HIV-AIDS	12	12	100
*Adansonia digitata*	Management of HIV-AIDS	9	12	75
*Geigeria aspera*	Stomach related infections	18	18	100
*Tragia dioica*	Stomach related infections	8	8	100
*Solanum aculeastrum*	Stomach related infections	12	12	100
*Solanum mauritianum*	Stomach related infections	21	21	100
*Solanum supicum*	Stomach related infections	6	6	100
*Securidaca longipedunculata*	Aphrodisiac for men	20	29	69
*Hypoxis haemerocallidea*	Aphrodisiac for men	10	20	50
*Cannabis sativa*	Vaal sick	7	11	64
*Tallinum caffrum*	Heart related infections	6	6	100
*Schotia brachypetala*	Diarrhoea	6	6	100
*Strychnos spinosa*	Diarrhoea	6	8	75
*Acacia karoo*	Diarrhoea	6	6	100
*Urginea sanguinea*	Blood purifier	7	19	37
*Jatropha erythropoda*	Blood purifier	10	10	100
*Withania somnifera*	Gynaecological complaints	14	14	100
*Ipomoea albivenia*	Gynaecological complaints	6	6	100
*Securidaca longipedunculata*	Headache	9	29	31
*Cannabis sativa*	Headache	4	11	36
*Neorautanenia mitis*	Footache	12	12	100
*Nerium oleander*	Toothache	2	4	50
*Sida cordifolia*	High blood pressure	6	6	100
*Vitis vinifera*	High blood pressure	3	5	60
*Azanza gackeana*	Painful joints	3	3	100
*Kirkia acuminata*	Fractured bones	4	4	100
*Aloe marlothii*	Ethno-veterinary infections	3	6	50
*Urginea sanguinea*	Ethno-veterinary infections	7	19	37
*Cassia abbreviata*	Diabetes	3	18	17
*Pterodiscus kellerianus*	New born babies	18	18	100
*Ehretia rigida*	New born babies	7	7	100
*Grewia flavescens*	New born babies	6	8	75
*Jartoha zeyheri*	Eye infections	14	26	53
*Elephantorrhiza burkei*	Eye infections	10	36	27
*Ximenia americana*	Asthma	4	11	36
*Dichrostachys cinerea*	Vomiting	4	7	57
*Gymnosporia senegalensis*	Vomiting	4	15	27
*Cynodon dactylon*	Tonsillitis	3	3	100
*Ziziphus mucronata*	Tonsillitis	3	4	75
*Catharanthus roseus*	Skin infections	7	11	63
*Terminalia sericea*	Skin infections	10	23	43
*Aloe marlothii*	Skin infections	3	14	21
*Gardenia volkensii*	Chest complaints	3	4	75
*Cassia abbreviata*	Anti-poison	3	18	17
*Blepharis diversispina*	Deceased’ wife	23	29	79
*Cucumis hirsuta*	Deceased’ wife	26	26	100
*Ziziphus mucronata*	Sores and wounds	1	4	25
*Carissa edulis*	Sores and wounds	3	13	23
*Peucedanum sulcatum*	General medicine	11	11	100
*Ipomoea alba*	General medicine	11	11	100
*Ipomoea spp*	General medicine	3	4	75

### Combination studies and plant species with Frerequency index ≥70

Eight medicinal plants species such as *Elephantorrhiza elephantine*, *Waltheria indica*, *Securidaca longipedunculata*, *Blepharis diversispina*, *Peltophorum africanum*, *Cissus quadrangularis*, *Sclerocarrya birrea* and *Elephantorrhiza burkei* reported FI value ≥70 hence have some pharmacological activities reported from literature (Table 6). About 12 combinations of medicinal plants species have been recorded in the current study (Table 7). *Waltheria indica* appeared in six of the 12 combinations, accounting to 50% and is used in the treatment of stomach ache, sexually transmitted infections, infertility, diarrhoea and strengthening of immunity in new born babies.

**Table 6:** Reported biological activity of the plant species with FI value ≥70

Plant species	Relevant Biological activities reported by other authors	References
*Blepharis diversispina*	None reported	None Reported thus far.
*Sclerocarrya birrea*	Analgesic, anti-inflammatory, antimicrobial, anti-proliferative, anti-oxidant, pro-apoptotic, anti-diarrhoeal,	[54–57]
*Elephantorrhiza burkei*	Anti-microbial, Anti-inflammatory;	[37, 45]
*Peltophorum africanum*	Anti-HIV, antimicrobial, anti-diabetic, anthelmintic,	[58, 59]
*Waltheria indica*	Antimicrobial, Antioxidant, anti-malarial, antiviral, antidiarrheal, analgesic anti-inflammatory	[60, 61]
*Securidaca longipedunculata*	Antimicrobial, anti-malarial, anthelmintic, anti-inflammatory, anti-diabetic, anti-oxidant, anti-parasitic	[62]
*Cissus quadrangularis*	Antimicrobial, Antioxidant, anti-malarial, antiviral, antidiarrheal, analgesic anti-inflammatory	[63]
*Elephantorrhiza elephantina*	Antimicrobial	[37]

**Table 7:** Reported combinations of various plant species in treating infections

Combination number	Main Medicinal plants	Other medicinal plants added	Condition treated	Mode of administration
1.	*Peltophorum africanum*, stem bark	A handful of *Elephantorrhiza burkei* roots, *Cassia abbreviata* stem bark, three nodes of *Cissus quadrangularis*	Dropsy and other STIs on a patient without sores	The mixture is cooked in 2 L of tap water in a clay pot and the patient have to inhale the heat coming out of pot for three consecutive days.
2	*Elephatorrhiza elephantina*, roots	A handful of *Jatropha zeyheri* root bark.	Eye infections	The two plant specimen are immersed in about 500 mL water and the resulting solution is used to wash eyes until healed.
3.	*Melia azeadarach*, Leaves	A handful of *Carpobrotus eludis* leaves and *Catharanthus roseus* leaves	Shingles	The leaves of the three plant species are chopped and added into a bath with mild water and the patient is washed for three consecutive days, three times a day or until the reddishness subsides.
4.	*Cassia abbreviata*, stem bark	A handful of *Elephantorhiza burkei* roots and *Catharanthus roseus* roots	Generally used to treat sexually transmitted infections.	The mixture is cooked in 1 L tap water and a full cup is taken orally, along a ground *Peltophorum africanum* stem bark, until the infection heals completely.
5.	*Cassia abbreviata,* Stem bark	A handful of *Blepharis diversispina* roots, *Elephantorrhiza burkei* roots, *Jatropha zeyheri* roots, *Cissus quadrangularis* and *Peltophorum africanum* stem bark	Generally used to treat sexually transmitted infections.	The plant materials are cooked in a 2 L water and half a cup of the resulting solution is drunk three times a day until the infection heals completely.
6.	*Cassia abbreviata,* Stem bark	*Pollichia campestris* roots, “*Matshilana”* roots, *Waltheria indica* roots and a handful of the “*Pitsa ya badisha”* bulb	Sexually transmitted infections and opportunistic infections.	The plant materials are cooked in about 3 L water and two cups are taken daily
7.	*Punica granatum*, Roots	*Hapargophythum procumbens* roots, *Waltheria indica* roots	Diarrhoea	The mixture is cooked in a 3 L bottle, and one cup is taken along the dried and ground fruit powder from *Punica granatum*.
8.	*Waltheria indica*, Roots	A handful of *Senna italica* roots, *Ipomoea albivenia, Hapargophythum* procumbens, *Peltophorum africanum* stem bark and one small cut of *Cissus cornifolia* bulb	Infertility	The mixture is cooked in a 2 L tap water and half a cup of the resulting tea like solution is drunk twice a day, treating infertility.
9.	*Waltheria indica*, Roots	A handful of various *Solanum* species, *Geigeria aspera* and *Senna italica* roots	Stomach aches and diarrhoea	The mixture is cooked in 2 L tap water and half a cup of the resulting solution may be drunk as often as possible, until the condition is treated.
10.	*Grewia flavescens*, roots	A handful of *Waltheria indica* roots, *Pterodiscus kellerianus* roots, “*Matshilana*” roots, *Senna italica* roots and any three different *Solanum* species roots	New born meal that strengthen the immunity and general growth of new born babies.	The plants are cooked in a 3 L tap water and the resulting solution is generally called ‘disha’ and is sucked by babies in a milk bottle.
11.	*Ipomoea bolusiana*, bulb	A handful of and *Cissus cornifolia* and *Pollichia campestris.*	Foot ache	The mixture is cooked in a 3 L clay pot, inhaled while still hot. When the heat cools off, the resulting mixture is poured into a bin and then used to wash the legs. The procedure is only done in the evening or during the night, once a day until the pain and infection heals.
12.	*Schotia brachypetala.*	A handful of *Psidium guajava* roots and *Dovyali*s spp	Diarrhoea	The plant materials are cooked in a 2 L water and a full cup of the resulting solution is reacted with half a spoon of ground seeds of *Punica granatum*. The solution is mixed and then taken orally three times a day until diarrhoea subsides.

## Discussions

### Demographic information and diversity of use of plant species

Traditional knowledge is mainly transferred from one generation to the next through mouth and such information may evacuate and disappear for good with time or becomes limited as life evolves [29, 30]. The demographic information of selected informant’s data shows that males (55%) dominates in the traditional knowledge compared to 45% of females. Contrarily, other authors reported the females to dominate in the traditional knowledge [31, 32].

The families such as Fabaceae and Malvaceae are dominant in the current study, reporting 14.63 and 8.54% respectively. The dominance of the Fabaceae has also been reported several times in ethnobotanical surveys at different localities [33] world-wide. The use of the branches, sap and seeds were all reported to be much lesser. In the current study, the use of the underground plant part contributes (58.6%), while stem bark reported 13.1%. The use of underground, stem bark and whole plant (especially herbs which are uprooted) is of major concern as it is extremely detrimental to the health of the plant species and may lead to plant species extinction.

### The informant consensus agreement

The technique is designed to highlight medicinal plant species that have a healing potential for a specific major illness. The plant species in major disease category, with F_IC_ values of 1 or very close to 1 indicate a high rate of informant consensus on plant species used against the major specific illness [28]. In the current work, the plant species used in the treatment of joints, fractured bones, anti-poison, aphrodisiac, chest complaints, tonsillitis, asthma, vaal-sick and toothache reported F_IC_ values of 1. A similar trend has been observed elsewhere in other countries [34, 35]. However, it should be noted that the number of species in the above mentioned ailment categories is also equivalent to1.

### Fidelity levels (FL) of the preferred medicinal plant species

Fidelity level is designed to reveal the percentage of informants claiming the use of a certain plant for the same purpose [36]. FL values of documented plant species are reported in Table 5.

In the current study, about 25 species revealed FL value of 100% against variety of diseases, suggesting that the informant’s state of knowledge is common when it comes to the uses of such plant species. Although *Mimusops zeyheri* and *Raphionacme hirsuta* revealed FL value of 100%, there is no data in the literature supporting the pharmacological effect of such species against pathogenic strains belonging to the traditional sphere of sexually transmitted infections.

It should also be noted that three plant species, such as *Bauhinia galpinii*, *Elephantorrhiza burkei* and *Cassia abbreviata*, from family Fabaceae appeared as some of the preferred plant species used against sexually transmitted infections, eye infections and as anti-poison respectively. Furthermore, *Peltophorum africanum*, *Eephantorrhiza elephantine*, *Elephantorrhiza burkei* and revealed frequency index (FI) values of 78, 85 and 90 respectively (Table 2). These data suggests that the family Fabaceae is generally important and used in the treatment of various human and animal infections. Although *E. burkei* in the current study is preferred to treat eye infections, it was also reported in the treatment of diarrhoea within other Bapedi groups [37]. These difference may well suggest that the traditional knowledge on use of plant species in the treatment of infections may differ from one locality to the other. Although the current work revealed most preferred species used in the treatment of various pathogenic infections, the biological activity of such medicinal plants still needs to be explored and verified experimentally. Furthermore, the plant species with high FL values are of greater importance in treating the related human and animal infections from the study site.

### Plant uses and ailments treated

The plant species reported in the current study are mostly used for treatment of human and animal infections while others are used for magical purposes. The most reported plant species are used in the treatment of sexually transmitted infections (24) followed by those used in the management of HIV-AIDS related infections (15), stomach ache (14) and plant species used in the treatment of ethno-veterinary infections (9). These results agrees with those of Peltzer et al., [38] who reported sexually transmitted infections to be mostly encountered and treated by African traditional healers. Amazingly, only a single plant each is reported to be used to treat pulmonary infections, mellitus diabetes and asthma.

Out of all the named medicinal plants in our survey, *Gardenia volskensii* is the only plant species reported to treat pulmonary related infections including tuberculosis. However, some of our informants revealed that for such purposes, bones from the chest of the Ostrich and nest of a dove “*leeba*” are chopped together and then administered to the patient. We found this difficult to validate scientifically as the doves may use different plant materials to build the nest and the age and gender of the ostrich was not identified in any of our informants. *Solanum* species are used to treat stomach related illnesses. One of our informants revealed that a mixture of a variety of *Solanum* species is the perfect solution to various stomach disorders and further used a name “Merolanarolana” referring to variety of such species when hiding the prescription form the patients. It should be noted that from the multi-purpose plant species reported, 28% species bears fruits and are identified as food plants as well. According to our informants, the use of the species as foodstuffs is not very important as there are no markets for such fruits within the study sites. However, the fruits are used as addition to foods within families and also eaten by boys when shepherding the cows on the mountains. The treatment of infections is more important than the food value. For the purpose of food, the indigenous people are reliant upon the agricultural crops such as maize, wheat, potatoes and leafy vegetables which are grown mainly during the summer season.

### Magical and ethno-veterinary plants species

**Fig. 5:**
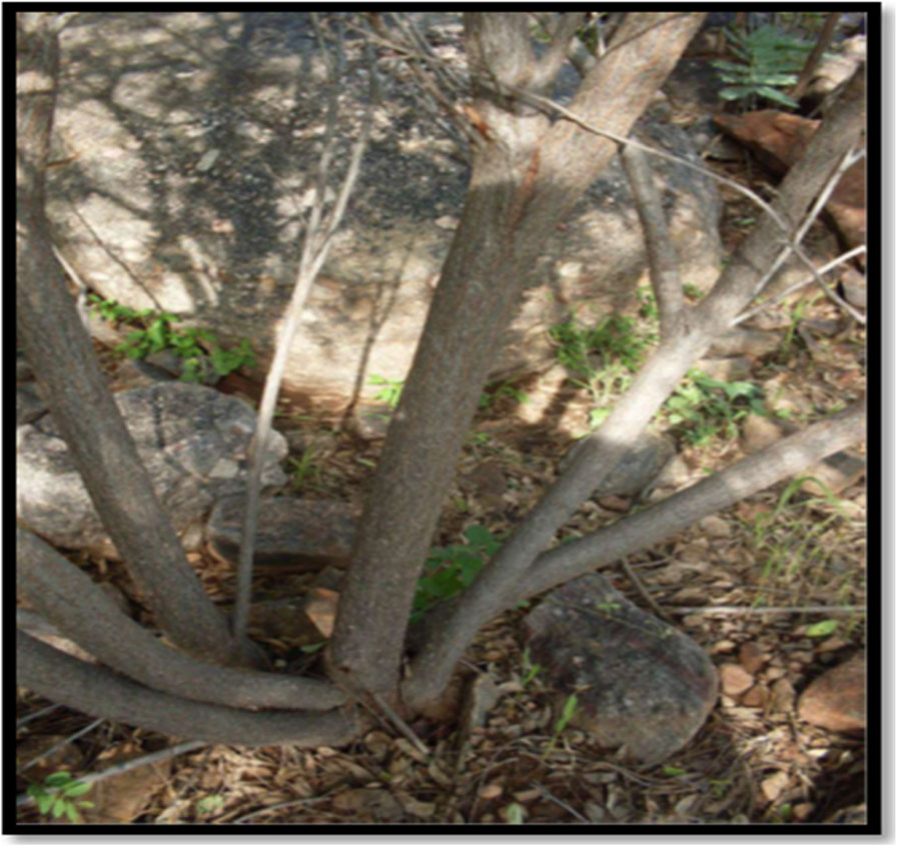
Multi-stemmed *Cassia abbreviata*

Out of 82 plant species reported in the study, about 12 plants are used for magical purposes, while 9 species are used in the treatment of various ethno-veterinary infections. *Sarcostema acidium* and *Cassia abbreviata* are the most reported magical plant species with frequency index of 58 and 45 respectively (Table 2), while *Elephantorrhiza burkei* and *Elephathorrhiza elephantina* are preferred for ethnoveterinary use with frequency index of 90 and 85 respectively. Plant species reported within this category are believed to be used to doctor homesteads there by protecting them from lightning, dispel the witches, returning some illnesses and calling upon some ancestral spirits. *Cassia abbreviata* is used for many other uses in various communities. However, the Pedi tribe use the multi-stemmed species mostly in the doctoring of homesteads. The multi-stems (Fig. 5) are believed to symbolise the number of huts in the family that might comprise of extended family members and a number of wives belonging to one husband [39].

### Food plants

Out of 82 plant species, 23 plants (28%) bear fruits are identified as food plants. *Strychnos madagascariensis* and *Psidium guajava* reported the highest FI value of 58, each while *Azanza garckeana* reported the lowest FI value of 5. Our current report corroborate that of Musina and Maroyi [40] who reported species such as *Scleorcarrya birrea*, *Mangifera indica*, *Psidium guajava*, *Punica granatum* and *Vanguera infausta* being used as food plants within Capricorn District, Limpopo Province. According to our knowledge, *Cissus cornifolia* was reported the first time within the country as a food source. However, it should be noted that the ethnobotanical survey of both the domesticated and wild edible fruits as sources of food within the Province is lagging behind and still needs to be enormously explored.

### Significance of names of plant species

Some plant species in the study are named either according to their physical morphological features, growth form or their role in the traditional indigenous medicine. *Urginea sanguinea* is commonly known as “Sekanama” which means “like meat” referring to the blades from the bulb of the plant species and its reddish colour. *Ximmenia caffra* is called “Motshidikgomo”. *Ximmenia* species are generally called “Motshidi”, while the word “kgomo” means cow, which a symbol of a bigger material or object is referring to the size of the fruit of species which is bigger than other *Ximmenia* species. *Hypoxis haemerocallidea* is known as “Monna wa maledu” referring to the beed-like structures protruding from the bulb and it translates to “a man with beed”. “Makgonatsohle” is a plant species which is generally used to treat all illnesses relating to stomach and it translates to “cure all” referring to the ability of the plant species to cure all illnesses. Although there is a general trend that all reddish medicinal plants are used to cleanse the blood.

“Thotamadi” is the name given to plant species and is generally believed to cleanse the bloodstream much better than all other species. “Madi” means blood. *Cissus quadrangularis* is indigenously known as “Mohlabadipoo”. The word “hlaba” means stabbing or pinching, referring to the pinching-like feeling that a patient generally feels after fumigation of the plant species. *Waltheria indica* is known as Mokhutesela, refereeing to the ability of the plant species to cool the stomach. “Khuta” means heals or stops the roaring or ripens. *Asparagus exuvialis* is the plant species which the indigenous family that have a function at home normally burns to disperse the clouds that may cause rain when there are blackish or dark clouds which are associated with evil spirits. The idea is to let the rain come back at a later time interval. “Phatlalatsa” means disperse while “maru” refers to clouds.

*Capobrotus eludis* is indigenously called “tima” which means cooling off, referring to the ability of the plant species to cool off the pain, heat and fever associated with shingles, which is also known as “belt” (*lepanta*). *Senna italica* is commonly called “Morotelatshotshi”. In Sepedi, the word “tshotshi” refers to ants, while “moroto” means urine, which generally refers to the yellowish colour of the resulting liquid after immersing the roots in water overnight. The yellow colour may be coming out of the root kernels which are light yellow when matured. The plant species grows in abandoned ploughing land and always have ants in close proximity everywhere it grows. Indigenous taxonomy therefore makes more sense to the traditional community than the scientific society.

### Mode of administration

In the current study, 73% of species are administered orally. The results in the current study corroborate that of other authors who reported the oral route as the most common mode of medicine administration [41, 42]. Besides *Securidaca longipedunculata* (root bark) which is taken along with mageu, all the medicinal plants species taken orally are cooked with tap water and drunk until the infections subsides or heal completely. *S. longipedunculata* is reported to be extremely bitter and have a lot of “after taste” and may at times result in vomiting. The use of mageu as a carrier assist in preventing such circumstances. Elsewhere, the root bark from *S. longipedunculata* is mixed with that of *Zanthoxylum humile* and taken with soft porridge to treat erectile dysfunction [43].

### Frequency index of documented plant species

Except *Blepharis diversispina*, all the species are reported to possess a potent antimicrobial activity against a variety of pathogenic microbial strains. In a way, the results in our current study validates the affectivity of various plant species against patahogenic microbial strains. However, it is amazing that the biological activity of extracts and isolated compounds from *B. diversispina* are not explored.

Medicinal plants with the highest FI value have related ethnobotanical uses in other cultures. For example, *Peltophorum africanum* and *Elephantorrhiza burkei* have been reported in the treatment of sexually transmitted infections, skin infections and diarrhoea amongst the Tswana, VhaVenda and Tsonga cultural groups and a potency on such activities have been reported as well [44–47]. These species are of vital importance in the treatment of reported infections in combinations as shown below (Table 7). *P. africanum* has also been implicated in the treatment of various ethnoveterinary infections [48–50].

### Combination studies of reported plant species

The combinations seems to be different from one traditional healer to the other. The purpose of compiling these combination studies was to assist the other researchers in selection of medicinal plant species relating to a specific illness. Earlier, [51], reported some different combination studies of related plant species, explaining that different traditional healers from different localities may use different plant species to treat different infections. The results in the current study shows that the traditional healers and plant sellers use variety of combinations in treating various ailments which includes sexually transmitted infections, eye infections, diarrhoea, and opportunistic infections associated with HIV-AIDS, new born babies illnesses and other gynaecological complaints as occurring in women. The other authors elsewhere reported the similar trend that indigenous systems use a combination of two or more plant species in treating infections [52]. However, from a scientific perspective, it may be difficult to determine which plant species contributes more active components than the others as there are a huge number of chemical compounds involved. However, these is generally believed to curb antimicrobial resistance.

### Domesticated plant species

From our visits in the homes of the informants, we found species such as *Withania somnifera*, *Ipomoea alba*, *Punica granatum*, *Carica papaya*, *Vangueria infausta*, *Sclerocarrya birrea*, *Kirkia acuminata*, *Cissus quadrangularis* and *Cassia abbreviata* as some of the plant species grown in at least 10 homes. However, other authors reported most of the plant species found homes as part of a garden to be used only as food supplements and ornamental plants [53]. In our study, some plant species such as *W. somnifera*, *C quadrangularis*, *K. acuminata* and *I. alba* are only used as medicine used to treat variety of human and animal illnesses. When asked why only those species are being domesticated, most informants believe that the plant species are used more often than others and are gradually declining in their natural environment. However, some healers believe that some plant species are believed to be efficient in treating infections only when collected from the wild. Such healers further believes that plant species in the wild are natural and have a stronger power that comes from gods and the wind.

## Conclusions

The traditional knowledge of the indigenous people of Blouberg varies from one traditional healer/ plat trader to the other. Traditional medicinal plants are mostly used in the treatment of human infections, especially sexually transmitted diseases, ethno-veterinary infections, as sources of food and for magical purposes. There is correlation in terms of ethnomedicinal use between cultures within Limpopo province. There is a need to explore the wild food plants as there is lack of data in that area of research. In the current, most plant species are used in the treatment of sexually transmitted infections, management of HIV-AIDS, stomach related infections and ethno-veterinary treatment. There is a need to further explore the possibility of documenting plant species used to treat such infections in future.
